# Supplementary material for: The feasibility of a web-based resilience-building program to prevent stress among danish pregnant nulliparous women: A randomised controlled feasibility trial
Source: PLOS Ment Health. 2025 Sep 8;2(9):e0000294. doi: 10.1371/journal.pmen.0000294 (PMC12798499; doi:10.1371/journal.pmen.0000294)
Supplement: Attachment — Submitted filename: Response to reviewers_140625.docx [file pmen.0000294.s002.docx]

# Response to Reviewers

Dear Editors and Reviewers,

Thank you for your thorough and constructive reviews of our manuscript entitled "The Feasibility of a Web-based Resilience-building Program to Prevent Stress among Danish Pregnant Nulliparous Women: A Randomised Controlled Feasibility Trial". We greatly appreciate the time and effort invested in evaluating our work. Please find below our point-by-point responses to the comments provided by both reviewers. All changes are marked in the revised manuscript using tracked changes.

On behalf of all co-authors,
Yours sincerely,
Monica Ladekarl

**Journal Requirements:**

| **Comment** | **Response** |
| --- | --- |
| Please provide separate figure files in .tif or .eps format. For more information about figure files please see our guidelines: https://journals.plos.org/mentalhealth/s/figures https://journals.plos.org/mentalhealth/s/figures#loc-file-requirements | We have now provided the figures in .tif format |
| . In the online submission form, you indicated that “By request”.  All PLOS journals now require all data underlying the findings described in their manuscript to be freely available to other researchers, either a. In a public repository, b. Within the manuscript itself, or c. Uploaded as supplementary information.  This policy applies to all data except where public deposition would breach compliance with the protocol approved by your research ethics board. If your data cannot be made publicly available for ethical or legal reasons (e.g., public availability would compromise patient privacy), please explain your reasons by return email and your exemption request will be escalated to the editor for approval. Your exemption request will be handled independently and will not hold up the peer review process, but will need to be resolved should your manuscript be accepted for publication. One of the Editorial team will then be in touch if there are any issues. | We have now inserted a paragraph in the manuscript explaining why the data cannot be made publicly available, except by special request (Page 34, lines 638–643):  Data from the intervention contains sensitive information and cannot be made publicly available for ethical and legal reasons. Public availability may compromise participant privacy, and this would not comply with Danish legislation ([www.datatilsynet.dk](http://www.datatilsynet.dk/)). Access to the data requires an application submitted to and subsequently approved by the steering committee. Data requests may be sent to Professor Berit L. Heitmann ([Berit.Lilienthal.Heitmann@regionh.dk](mailto:Berit.Lilienthal.Heitmann@regionh.dk)) or the Reseach Unit for Diet and Health at The Parker Institute ([bfh-eek@regionh.dk](mailto:bfh-eek@regionh.dk)). |
| Please review your reference list to ensure that it is complete and correct. If you have cited papers that have been retracted, please include the rationale for doing so in the manuscript text, or remove these references and replace them with relevant current references. Any changes to the reference list should be mentioned in the rebuttal letter that accompanies your revised manuscript. If you need to cite a retracted article, indicate the article’s retracted status in the References list and also include a citation and full reference for the retraction notice. | Thank you for the reminder. We have reviewed our reference list carefully, and to the best of our knowledge, none of the cited studies have been retracted. Should we have overlooked anything, we would of course be happy to correct it upon notification. |
|  |  |

## Reviewer #1

| Reviewer #1: The current paper describes a feasibility/pilot trial for web-based Resilience to prevent stress among pregnant nulliparous women in the Danish population. The study has many strong points, 1. Being a feasibility study no power analysis is attempted, rather sample size is based on feasibility 2. The results correctly do not include any statistical test or p-values 3. The emphasis is on the feasibility of recruitment, randomization, retention, etc. I find the results reported have confidence intervals which is also appropriate. Some more clarification could be helpful, such as: | |
| --- | --- |
| **Comment** | **Response** |
| As per the CONSORT diagram, the difference between screening and randomization is unclear. It seems only one subject is lost between the two phases. If true this is remarkable and needs more discussion. | We agree that the small attrition between information and randomisation may appear unusual. However, this reflects the structure of our recruitment strategy rather than an exceptionally high enrolment rate. We applied a broad and feasibility-focused approach: participants were informed orally and via leaflets distributed in antenatal clinic waiting rooms and during pregnancy classes. As part of the feasibility assessment, we recorded the total number of women informed (n=1054), even though many would not be eligible or interested.  We did not have permission to access electronic health records for pre-screening; therefore, we could not pre-select only eligible participants. Instead, potential participants self-screened based on the inclusion criteria listed in the leaflet. Consequently, only women who found the study relevant and believed themselves eligible contacted us to participate. A few exceptions were registered (e.g., BMI slightly above the limit). This strategy resulted in low exclusion numbers after contact and explains the apparently small loss between information and randomisation in the CONSORT diagram  (Pages 14-15, lines 310-312 - revised flowchart - and lines 319):  The number of women informed about the study reflects ‘broad feasibility-focused recruitment’ via oral information and distribution of leaflets in antenatal clinics and pregnancy classes. Inclusion criteria were clearly stated in the leaflet, and potential participants self-referred only if they believed themselves eligible. This approach, combined with the absence of electronic pre-screening, resulted in low exclusion numbers post-contact. |
| It is stated, in conclusion, that some changes are needed in the large-scale RCT based on what is learned from the feasibility trial. This must be stated more elaborately as this is the primary reason for conducting any feasibility trial | We agree and have now elaborated on the changes suggested for a full-scale RCT. This includes adapting the intervention format, modifying reminders, improving individual tailoring, and refining inclusion strategies. This is outlined in the Discussion and Conclusion sections (Pages 32, lines 603-608):  “Based on the findings of the feasibility study, we propose the following specific changes before launching a full-scale RCT: (1) Reducing questionnaire burden and adapting all formats for mobile use; (2) Minimizing perceived pressure by reducing reminder frequency and allowing customization; (3) Using GDPR-compliant platforms to track individual usage and improve data quality; (4) Involving pregnant women directly in tailoring the intervention to ensure relevance and feasibility; and (5) broaden the target group to also include pregnant women with known stress.” |
| Since the trial cannot be blinded to the subject its implication on the subject-level bias needs further attention. | Thank you for this important point. We have now included a discussion on the lack of blinding and the risk of expectancy bias in the Limitations section. We also address how future designs might mitigate these effects (Page 31, lines 564-570):  “Due to the nature of the intervention, blinding of participants and study staff was not possible, which may have introduced expectancy or performance bias, particularly for self-reported outcomes such as perceived stress. This is a known challenge in behavioural intervention trials, where active blinding is often not possible (52). However, except for standardised reminder emails and the option to attend a one-time information session, there was no direct contact with the intervention group during the study period, limiting the risk of differential attention bias. To decrease bias in future studies, we will consider blinding outcome assessors and employing an active comparator to balance expectancy effects across groups.” |
| While statistical testing is not needed some aspect of statistical modeling will be helpful, which is completely missing from the manuscript. | We appreciate the suggestion. We have now clarified in the Methods and Results and Limitations that although no hypothesis testing was planned, we included exploratory adjusted mean differences with confidence intervals for key outcomes to offer a preliminary indication of potential effects. These adjustments are now more clearly explained (Page 13, lines 291-295):  “As this was a feasibility study, no formal hypothesis testing was conducted. However, to explore potential trends, we conducted simple linear regression analyses to estimate adjusted mean differences with 95% confidence intervals between groups. These were adjusted for baseline values of the corresponding outcome measures. The aim was not to infer effectiveness but to inform the design and sample size estimation for a future RCT.”  (Page 13, lines 291-295):  Table 4 presents exploratory adjusted mean differences between intervention and control groups in gestational week 35, based on simple linear regression analyses controlling for baseline values of the corresponding outcomes. For example, the adjusted mean difference in Perceived Stress Scale (PSS) score was –3.26 (95% CI: –5.55; –0.96), indicating a statistically significant and potentially meaningful reduction in perceived stress in the intervention group. Similarly, the adjusted difference in resilience (CD-RISC) was +3.39 (95% CI: 0.64; 6.14), indicating a potential improvement in participants’ ability to cope with stress.  Due to the small sample size and the feasibility design, these findings should be interpreted with caution. However, the results suggest favorable trends across several mental health outcomes, including perceived stress, depression, and anxiety, although confidence intervals often included the null. For maternal weight-related outcomes, postpartum weight retention (PPWR) favored the intervention group with an adjusted difference of –1.59 kg (95% CI: –3.29; 0.11), but were not statistically significant, while no effects were observed for birth weight or gestational age. At the two-month postpartum follow-up, no group differences were observed (data not shown).  (Page 31, lines 584-586):  “Although the study was not powered to assess effectiveness, the exploratory adjusted estimates may still inform future sample size calculations and provide preliminary insights into possible effect sizes.” |

## Reviewer #2

| Reviewer #1: This was a very interesting and well written article. The study looked at the Feasibility of a Web-based Resilience-building Program to Prevent Stress among Danish Pregnant Nulliparous Women. The title of the article matches the information discussed in the article. The abstract and introduction of the study shows that authors have thought this research through. They also provided enough detail relating to their methods and the decisions surrounding their chosen methods.  Even though their article was well written, there are potential areas of improvement: | |
| --- | --- |
|  | |
| **Comment** | **Response** |
| The first area that I would encourage authors to strengthen is their discussion. The discussion felt like a repeat of the methods section or something that should be fused with the methods section. It did not discuss the second part of your topic (ie preventing stress) but was more on the technicality of the findings. I was looking forward to hearing authors’ debates and perspectives on the ‘did the program build pregnant women’s resilience to stress’, especially because authors presented their findings very well. Situating the discussion within existing research would also benefit the study. | Thank you for this observation. We have tried to make the Discussion section focus more clearly on the potential effect of the intervention on stress and resilience, including participant feedback and integration with existing literature (Pages 26-27, lines 452-461):  Psychological stress during pregnancy is a significant public health concern due to its potential adverse effects on maternal well-being and birth outcomes. Despite increasing interest in digital mental health support, evidence for stand-alone digital interventions designed to prevent stress or build resilience in pregnant women remains limited (22). Our study addresses this gap by evaluating the feasibility and preliminary impact of *MyResilience*, a web-based resilience program adapted for pregnant women.  Although the study was not powered to assess effectiveness, our exploratory analyses suggested modest improvements in perceived stress and resilience among participants. This aligns with previous research showing that digital tools teaching mindfulness, acceptance, and cognitive coping strategies can improve emotional regulation and reduce stress, both generally and during pregnancy - including potential downstream benefits for infant outcomes (18,21,40–43).  (Pages 27, lines 463-474):  Taken together, our findings suggest that the intervention may hold promise in helping pregnant women prevent or better manage stress, though this remains to be confirmed in a larger trial. While the modest reductions in perceived stress and increases in resilience were exploratory, they are noteworthy considering the relatively low intensity of the intervention and the high baseline resilience in our sample. However, the mixed user experience also underscores that perceived relevance and timing of such interventions are critical to their effectiveness. For example, participants who reported benefiting from the tools often described using them proactively in everyday stress situations, suggesting that the intervention may have supported their ability to cope with stressors as they arose—core to the concept of resilience. In contrast, women who did not perceive themselves as stressed were less likely to engage. Future studies should more closely investigate how individual differences in baseline stress, coping styles, and readiness to engage affect the uptake and effect of resilience-building interventions during pregnancy. |
| Please also add more detail on how you analysed your telephone interviews. Provide more information on the data analysis method used and the decisions you took to eventually get to your themes. | We have now expanded the section on qualitative data analysis to explain how the predefined matrix was used and how themes were derived from the interview data (Pages 12, lines 276-278):  The guide included questions such as “Which exercises do you remember?” and “What were your impressions of the website and the reminders?”, aiming to explore participants’ experiences in more depth.  (Pages 13, lines 299-302):  We used a deductive content analysis approach based on Elo & Kyngäs (39)**,** applying a predefined coding matrix focusing on form, content, and execution. Two researchers coded the interview transcripts and resolved discrepancies through discussion. The emerging themes and categories were mapped to the predefined matrix. |
| Your conclusion can also be improved too so that the article ends with the same intensity that you introduced it with. | We agree and have now elaborated on the changes suggested for a full-scale RCT. This includes adapting the intervention format, modifying reminders, improving individual tailoring, and refining inclusion strategies. This is outlined in the Discussion and Conclusion sections (Pages 32, lines 603-608):  “Based on the findings of the feasibility study, we propose the following specific changes before launching a full-scale RCT: (1) Reducing questionnaire burden and adapting all formats for mobile use; (2) Minimizing perceived pressure by reducing reminder frequency and allowing customization; (3) Using GDPR-compliant platforms to track individual usage and improve data quality; (4) Involving pregnant women directly in tailoring the intervention to ensure relevance and feasibility; and (5) broaden the target group to also include pregnant women with known stress.” |
